# Acknowledgement to Reviewers of *Genes* in 2017

**DOI:** 10.3390/genes9010033

**Published:** 2018-01-11

**Authors:** Genes Editorial Office

**Affiliations:** MDPI AG, St. Alban-Anlage 66, 4052 Basel, Switzerland

Peer review is an essential part in the publication process, ensuring that *Genes* maintains high quality standards for its published papers. In 2017, a total of 389 papers were published in the journal. Thanks to the cooperation of our reviewers, the median time to first decision was 24 days and the median time to publication was 56 days. The editors would like to express their sincere gratitude to the following reviewers for their time and dedication in 2017:
Abdelmohsen, KotbLibault, MarcAbdulkadir, Sarki A.Lifjeld, Jan T.Abou-El-Enein, MohamedLim, Sun-HyungAbu-Romman, SaeidLin, Ting-NienAfasizhev, RuslanLindsay, A. J.Agnelli, LucaLing, HuiAhlenstiel, ChantelleLiu, BinAhnn, JoohongLiu, ChangningAkbarian, SchahramLiu, Chao-LinAkerman, IldemLiu, Cheng-HsienAkkari, AnthonyLiu, Cheuk LunAklilu, BehailuLiu, DexiAleman, FernandoLiu, FanAleman, Tomas S.Liu, GangAlhawarat, MohammadLiu, HaipeiAlifano, PietroLiu, HudanAlkowni, RaedLiu, JianquanAllers, ThorstenLiu, LijunAlmeida, PedroLiu, ShenkuiAlminana, CarmenLiu, WeizhenAlonso, JavierLiu, ZhihuiAlpay-Karaoglu, SengulLloyd, R. StephenAlvarez, Juan B.Löffler, MarkusAmar, LaurenceLogue, Mark W.Amodeo, AmandaLong, DavidAnderson, Robert E.Lönnberg, TapioAndersson, Jan O.Lopes, Fabrício MartinsAndolfo, GiuseppeLópez De Heredia, UnaiAndrade, ArturoLópez-Baena, Francisco JavierAngelis, Karel JanLoreni, FabrizioAnglani, FrancaLorenz, BirgitAngrand, Pierre-OlivierLorite, PedroAniello, FrancescoLove, Donald R.Antoniadi, IoannaLu, GangAo, PingLucchesi, PaulaArcidiacono, BiagioLudwig, MichaelArenas Busto, MiguelLudwig, NicoleArno, GavinLuebeck, GeorgArrighi, Jean-FrançoisLyubchenko, YuriArroyo Pardo, EduardoMacDonald, Clinton C.Arsenian-Henriksson, MarieMacDonald, IanArtemenko, YuliaMacDonald, JessicaArtero, RubenMaciejewska, BarbaraAtanassova, RossitzaMacovei, AncaAtzmon, GilMacrander, JasonAuburger, GeorgMadesis, PanagiotisAyer, DonaldMaeda, AkikoAyers, DuncanMaffeis, ClaudioAzevedo, HerlanderMafi Moghaddam, SamiraAzuma, MizukiMaggo, SimranAzuma, YumikoMagor, BradleyBaarends, WillyMai, SabineBachem, Christian W.B.Majello, BarbaraBajpai, RichaMakarevitch, IrinaBakay, MarinaMakino, HiroshiBaker, Michael E.Małgorzata B, ŁobockaBakker, F.T.Mamuris, ZissisBakkeren, GuusManceau, MarieBalcells Ortega, IngridManova, VasilissaBalestrazzi, AlmaMaracaja-Coutinho, ViniciusBallizany, WouterMarchetti, MartaBalloy, VivianeMarcos, MiguelBandini, ErikaMargres, MarkBanerjee, KalpitaMariadason, John M.Bano, DanieleMaritschnegg, ElisabethBarabas, PeterMarkey, Michael P.Barac, AnaMarques, NatáliaBarbolina, Maria V.Marsola Giroti, AndréBarlow, JacquelineMartegani, EnzoBártolo, InêsMartens, J.H.A.Basturea, GeorgetaMartikainen, RiikkaBatchu, RameshMartin, MatthiasBates, DavidMartin, NickBatut, JacquesMartín-Durán, Jose MaríaBazire, AlexisMartinez García, EstebanBecker, AnkeMartínez, ConstantinoBednarek, PiotrMartinez, ManuelBeierle, ElizabethMartínez-Espinosa, Rosa MaríaBelles, XavierMartinez-Nunez, RocioBentivegna, AngelaMartins De Oliveira, Paula AlexandraBenvenuto, ChiaraMartin-Vasallo, PabloBen-Yosef, TamarMastrangelo, Anna M.Berardi, AnnaMatarese, Laura E.Berchie, Joseph NketiahMatsunaga, SachihiroBerger, PhilippMatta, JaimeBerger, WolfgangMattarelli, PaolaBerindan-Neagoe, IoanaMatyasek, RomanBermejo, RodrigoMay, FelicityBertoni, BernardoMcCarthy, ElizabethBettaieb, AhmedMcDaneld, Tara G.Beukeboom, LeoMcElroy, KyleBhadauria, VijaiMcGraw, SergeBhattacharya, SoumyaroopMcintosh, RobertBi, XinMcQuillin, AndrewBianco, PieroMecocci, PatriziaBiasucci, GiacomoMegraw, Timothy L.Birks, JohnMelnyk, CharlesBirnbaum, Kenneth D.Menck, CarlosBissonnette, NathalieMenda, NaamaBitler, BenjaminMeng, DongBlackmon, HeathMereu, ElisabettaBlattner, ChristineMerla, GiuseppeBlaze, JenniferMessaritakis, IppokratisBock, Niko C.Metzler-Guillemain, CatherineBode, Robert F.Miao, XiangyangBolibok-Brągoszewska, HannaMica, EricaBolz, Hanno J.Michailidis, G.Bonilla González, EdmundoMiele, LucioBonnycastle, LoriMilani, LilianaBontemps, CyrilMillard, AndrewBorde, ValérieMills, DavidBose, DebojitMin, WangBouma, Gerrit J.Mingoia, MarinaBourgeois, CyrilMino, MasanobuBover-Arbos, PereMitoma, HirokiBoyd, ChrisMittal, GayatriBoye, Sanford L.Mizuno, KentaroBozzaro, SalvatoreMizuno, TooruBradley, DanielMody, VickyBramham, Clive R.Moe, LukeBrannick, Erin M.Moghaddar, NasirBraun, ThomasMohanta, TapanBreschi, AlessandraMolineris, IvanBright, Jo-AnneMolinier, JeanBritt, Anne B.Monforte, Antonio J.Broderick, ShaunMongodin, EmmanuelBrunet, FrédéricMontag, JudithBubulya, Paula A.Montanaro, LorenzoBuckleton, JohnMontecucco, AlessandraBudak, HikmetMooers, BlaineBuell, RobinMorales, JulioBuratti, EmanueleMoreno Switt, AndreaBusch, HaukeMoreno-Hagelsieb, GabrielBushakra, Jill M.Moretzsohn, MarcioCabral-de-Mello, DiogoMori, HirotadaCaeiro, MariaMorou, EvangeliaCaetano-Anolles, DerekMorphew, Russell M.Cai, YudongMorris-Rosendahl, DeborahCalabrò, ViolaMorse, DavidCaligo, Maria AdelaideMortiboys, HeatherCalixto, Cristiane P.Mosher, RebeccaCallis, JudyMota, João FelipeCalvo, Jorge H.Moulin, LionelCampbell, MichaelMounsey, KateCampos, EricMoura, Ana Silvia A. M. T.Cândido, ElizabeteMower, Jeffrey P.Cannon, StevenMueller, Niklaus H.Cantoral, Jesus ManuelMuhovski, YordanCaparros-Martin, JoséMuller, Patricia A. J.Caparrós-Ruiz, DavidMullin, Gerard E.Cappelletti, GraziellaMünsterberg, AndreaCarapito, RaphalMurakoshi, HidejiCarillo, PetroniaMurphy, Sean VincentCarlberg, CarstenMurphy, Sharon E.Carr, AntonyMurphy, ThereseCarraro, NicolaMurphy, TomCasati, PaulaMurray, AnnaCastanera, RaúlMychaleckyj, JosyfCastelo Branco Carvalho, Luiz JoaquimNadif Kasri, NaelCastiglia, RiccardoNagarajan, RadhaCastro, Pedro HumbertoNagase, HirokiČerná, MarieNagpal, RavinderCesco, StefanoNakagawa, TakuroChaix, RaphaëlleNakajima, MasatoshiChan, AgnesNakamura, ToruChan, Raquel L.Nam, Jin-WuChandler, John W.Namihira, MasakazuChang, SuhwanNanba, EijiChapman, Mark A.Narita, MasashiChatterton, ZacNazarov, PetrChaudhuri, JayantaNelissen, HildeChaves, RaquelNepal, MadhavChen, FengNettersheim, DanielChen, JianNikoh, NaruoChen, JianqunNishihara, M.Chen, Li-QingNishikawa, ToruChen, Rong-JaneNishimura, TaisukeChen, RuiNiu, ChenChen, ShaoliangNoble, Janelle A.Chen, Shu-ChuanNoghero, AlessioCheng, DeNomura, MikaChiang, John (Pei-Wen)Noren Hooten, NicoleChiang, Yu-ChungNorthcross, Amanda L.Chişamera, GabrielNowicki, MarcinChiurazzi, MaurizioNúñez De Cáceres González, Francisco F.Chung, EuiryongNunney, LeonardCiaramella, MariaNuthikattu, SaivageethiCimmino, AmeliaO’Connell, MatthewCiudad, Carlos J.O’Connor, RebeccaClark, EmilyO’driscoll, MarkCochrane, Dawn R.O’Reilly, Paul F.Coelho, TracyOdierna, GaetanoCohen, DavidOehme, InaCole, JeffÖhrfelt, AnnikaColeman, William B.Okumura, Sei-ichiColetta, Dawn K.Olde Loohuis, LoesColli, Maikel LuisOlmo, EttoreCollins, CatherineOlson, Wilma K.Comander, JasonOlsson, SannaConnell, GregoryOltean, SebastianCopeland, NikkiOphir, RonCoppieters, FraukeOrtega, Francisco JoséCorella, DoloresOsada, MakotoCorrell, NathanOsbourn, AnneCosta, MarinaOzaki, ToshinoriCostanzo, VincenzoPaccanaro, AlbertoCoto, EliecerPaek, JungwookCotterill, SuePage, MelissaCournac, AxelPagliarani, ChiaraCoutinho, João PauloPál, MagdaCoutinho, Luiz LehmannPálsson, ArnarCowell, IanPalsson, Bernhard Ø.Cox, Robert H.Pan, MingguiCox, TimothyPanaccione, DanielCremer, MarionParadowska-Gorycka, AgnieszkaCronberg, NilsPardini, BarbaraCui, YanPark, Seon JooCuming, Andrew C.Parker, Stephen C.J.Czechowski, TomaszParvin, Jeffrey D.D' Souza, RenaPasantes, JuanDaczewska, MałgorzataPassos-Bueno, Maria RitaDaigaku, YasukazuPatil, BasavaprabhuDang, ChiPatil, DeepakDarbani, BehroozPavlov, YouriDardenne, EtiennePayne, AnnetteD'Argenio, ValeriaPazos, Antonio JuanDas, MalayPecinka, AlesDavies, KayPelicci, Pier GiuseppeDavuluri, RamanaPemberton, TrevorDay, JeremyPeng, XuDe Bello Cioffi, MarceloPenn, LindaDe Bortoli, MichelePérez-Montaño, FranciscoDe Castro Soares, SiomarPeterson, Erik J.De Castro, LianePetretto, Enrico G.De Martino, AndreaPicardi, ErnestoDe Masi, LuigiPickett, HildaDe Piccoli, GiacomoPickrell, AliciaDe Smet, Marc D.Piekiełko-Witkowska, AgnieszkaDe Vadder, FilipePilot, MalgorzataDe, KuntalPilyugin, MaximDean, CarolinePingault, LiseDebelius, JustinePinthong, KritDegrassi, FrancescaPiscitelli, AlessandraDekker, MarloesPita, SebastianDel Campo, RosaPiton, AmélieDelgado Valerio, PatriciaPiya, SarbottamDemuth, JeffPluquet, OlivierDen Hertog, JeroenPopovich, DavidDeng, JayPoschenrieder, CharlotteDeng, PengPrakash, AishwaryaDeng, WuminPraud, ChristopheDennis, JonProchownik, Edward V.Depamphilis, MelvinProsseda, GianniDeryusheva, SvetlanaProst, StefanDeyholos, MichaelPulvirenti, AlfredoDhondt-Cordelier, SandrinePuterman, EliDias, Guilherme BorgesQi, RongsuDickinson, Timothy A.Qiu, YiDickstein, RebeccaQiu, Yin-LongDing, BaoqingQuax, TessaDing, LinQuero, Grazia MarinaDion-Côté, Anne-MarieQuesada Pérez, Víctor ManuelDistel, MartinRaake, Philip W.J.Dluzen, DouglasRaanan, HagaiDong, JiaqiangRabinovitch, Peter S.Downie, J. AllanRadespiel, UteDrancourt, MichelRådinger, MadeleineDriscoll, James J.Rae, BenjaminDu, XiaoyongRajpurohit, SurendraDuan, ZhijunRamachandran, VinoyDubrez, LaurenceRamírez, LucíaDuffy, Aaron M.Ramírez, MarthaDufour, AlainRass, UlrichDumont, BethReuter, KlausDuncan, JacqueRey, LuisDunning Hotopp, JulieRhind, NickDutta, ArijitRibeiro-Barros, AnaDwyer, Andrew A.Ricci, EnzoDziewit, LukaszRichetto, JulietEar, JasonRiera, MarinaEbert, AllisonRimbara, EmikoEdae, Erena A.Ríos, Glenda LauraEdlich, FrankRiveiro Álvarez, Rosa MaríaEgamberdieva, DilfuzaRoberts, Thomas C.Egan, AshleyRobin, ArifElder, RhoderickRogakou, EmmyEl-Naggar, AdelRondanino, ChristineEndre, GabriellaRönnberg-Wästljung, Ann ChristinEnemark, EricRosa, Bruce A.Enguita, FranciscoRosani, UmbertoErgün, SercanRose, Laura E.Ericson, Per. G. P.Roselló, SalvadorEscher, PascalRosen, JeffreyEscudé, ChristopheRoss, KehindeEspeso, Eduardo A.Rosselló, Josep A.Eymin, BeatriceRothstein, RodneyFabris, LindaRoubelakis-Angelakis, KalliopiFalahati, HaniehRoussel, Martine F.Falahati-Anbaran, MohsenRu, SushanFan, XiuchengRubtsov, NikolaiFang, MeiyingRuden, DouglasFant, Jeremie B.Ruggeri, PaoloFarré, MartaRuíz, AgustínFauré, JulienRuiz-Herrera, AuroraFelling, RyanRuiz-Ruano, Francisco J.Feng, HuiRusso, AntonellaFeng, WenyiRusso, AntonioFeo, FrancescoRuzicka, William BradleyFerguson-Smith, MalcolmRyan, MarionFerreira, PedroSabaawy, Hatem E.Fierro, FranciscoSabina, HammerFigueroa-Romero, ClaudiaSacchi, Gian AttilioFill, Taícia PachecoSaghatelian, AlanFinlay, Graeme JohnSaha, SuryaFionda, CinziaSahu, JagajjitFischer, KatjaSakamoto, KeiFolco, Hernan DiegoSalehin, MohammadFopp-Bayat, DorotaSalentijn, Elma MjForcales, Sonia-V.Salvatore, FrancescoForrest, RachelSangild, Per TorpForterre, PatrickSantarpia, LiberoFosu-Nyarko, JohnSantiago, JoséFoulongne-Oriol, MarieSantiago, TashaFournier, GregorySantin, IzortzeFox, JonathanSantino, AngeloFozo, ElizabethSantoro, AureliaFraga, Mario F.Santos, RosárioFrancis, David M.Satoh, ShigeruFranssen, HenkSatou, RyousukeFrary, AnneScarlett, GarryFreischmidt, AxelScherer, GüntherFröhlich, Leopold F.Schlumbaum, AngelaFurey, TerrenceSchneider, SabineFusaki, NoemiSchneiders, ThamaraiGabel, HarrisonSchrenk, MatthewGabellini, DavideSchroeder, DanaGabriel, KyleSchutte, BrianGadaleta, AgataSchwartz, SchragaGadaleta, MarianaSchwarz, ErichGagos, SarantisSchweickert, AxelGahlaut, VijaySchwitzgebel, ValérieGalat, AndrzejSclavi, BiancaGalbiati, MassimoSears, Rosalie C.Galindo, AntonioSeashols-Williams, SarahGall, JosephSeckinger, AnjaGallant, PeterSecombe, JulieGallemí, MarçalSedlazeck, FritzGamage, Dilani G.Selvaggi, MariaGamble, MatthewSen, Taner Z.Ganopoulos, IoannisSenthil Kumar, NachimuthuGaranto, AlejandroSeo, Hak SooGarcia-Alonso, Luz M.Serino, GiovannaGarrido, DanielSeroussi, EyalGarrido-Ramos, Manuel A.Serretti, AlessandroGase, KlausServitja, Joan MarcGaulton, Kyle J.Shackelford, Rodney E.Geigl, Eva MaríaShanmugam, AvinashGerdol, MarcoSharma, HimanshuGibert, YannSharma, SudhaGibson, DeannaSharon, DrorGil, RosarioShavrukov, YuriGilbert, ThomasShen, ShihuaGill, Clare A.Shen, YulongGilson, EricShiina, TakashiGiordano, FrancescaShim, Sung HanGirdland Flink, LinusShnyder, SteveGitzendanner, MattShukla, SanjayGiudice, JimenaSieburth, Leslie E.Giuliani, AlessandroSiegele, DeborahGladyshev, EugeneSieving, Paul AlbertGleason, CynthiaSimpson, CraigGlover, ThomasSingh, KashmirGlover, Thomas W.Singh, RaviGmeiner, William H.Singh, ShivaGodler, David ESinha, KalyanGoedert, JamesSitia, GiovanniGoetzman, Eric S.Skibbens, RobertGogarten, PeterSkinner, Daniel Z.Golczyk, HieronimSkinner, MarkGoldammer, TomSkovgaard, OleGollin, Susanne M.Slavoff, Sarah AGomez, DavidŚliwka, JadwigaGondi, Christopher S.Smaczniak, CezaryGonzalez, AngelSmith, Colin P.Gonzàlez-Duarte, RoserSmith, DarrenGonzalez-Halphen, DiegoSmith, David I.Gorshkova, TatyanaSmith, Sinead M.Gosselet, FabienSomm, EmmanuelGoya, Gerardo F.Son, HyeonGraille, MarcSorci, GuglielmoGranberg, FredrikSpangler, Gordon L.Granot, DavidSpano, GiuseppeGranzier, Henk L.Spiro, StephenGraphodatsky, AlexanderSprent, JanetGreene, Catherine M.Srebrow, AnabellaGreene, Lesley H.St.Helen, GideonGregory, ChristopherStafforst, ThorstenGregory-Eaves, IreneStamm, StefanGrene, RuthStathopoulos, GeorgiosGriesenbach, UtaStear, MichaelGriffin, DarrenStephan, RogerGriffiths, GarethStepien, EwaGrifoni, DanielaStȩpkowski, TomaszGrimwade, JuliaStevenson, JanelleGrunau, ChristophStewart, JasonGrzebelus, DariuszStewart, RodneyGuerri, ConsueloStougaard, JensGuhathakurta, SubhrangshuStrano, SabrinaGuidotti, AlessandroStrom, Stephen C.Gulick, Patrick J.Stülke, JörgGupta, AnkitSu, JianqiangGupta, ShashiSuarez, Andrew V.Gurevich, VesovoldSubramani, MayavanGuyomarc'h, SoazigSucgang, Richard S.Guyot, RomainSudheesh, ShimnaHaak, DavidSuga, AkikoHadar, YitzhakSuga, HiroshiHadi, SibteSugimura, HaruhikoHagelberg, ErikaSugita, MamoruHampel, MiriamSun, XiaolinHan, LengSun, XinghuiHanikenne, MarcSun, ZhifuHansson, BengtSung, SibumHardiman, GarySunnerhagen, MariaHartl, MarkusSunseri, FrancescoHasanuzzaman, MirzaSuryawanshi, VasantikaHatakeyama, KatsunoriSussman, JoelHatch, NanSutherland, BenHatzis, PantelisSymonova, RadkaHauser, Michael ArthurSzakonyi, DoraHausner, GeorgSzczelkun, Mark D.Hayashi, TakaakiSzklarczyk, DamianHe, CaiyunTakahashi, TatsuroHe, JianqingTakano, AtsukoHegde, Muralidhar L.Takeshita, TatsuyaHeideman, WarrenTakezaki, NaokoHeller, Klaus-GerhardTakuno, ShoheiHenriksson, Marie ArsenianTamm, AguHenry, YvesTan, Ek HanHerbig, AlexanderTanabe, KatsuyukiHernandez, WenndyTang, JijunHerny, RobertTang, PatrickHerránz, HéctorTansey, WilliamHertweck, Kate L.Taoka, MasatoHeyne, HenrikeTaube, JoeHiller, N. LuisaTavladoraki, ParaskeviHirayama, TakashiTeeri, TeemuHizel, CandanTeige, MarkusHo, YeungTeixeira, CarlosHobza, RomanTemel, AslihanHoffmann, Michele J.Tennessen, Jacob A.Hofman, CourtneyTerrier, OlivierHolloway, JohnTerry, Randall G.Holsinger, DamianTerskikh, Alexey V.Holub, EricTétreault, Marie-PierHolzinger, AndreasTetz, VictorHorikawa, KazukiThomas, Elizabeth A.Hornsby, Angela D.Thompson, JeffreyHotta, YoshihiroThong, Melissa S. Y.Houben, AndreasThybert, DavidHowe, KerstinTian, BinHowitt, Crispin A.Tian, Chang-FuHsing, Yue-Ie C.Timmis, JeremyHu, JinguoTing, DavidHu, QiwenTiriveedhi, VenkataswarupHu, Yueh-ChiangTitorenko, Vladimir I.Hu, ZhiliangTodd, Peter K.Huang, Chao-ChengTokar, ErikHuang, Hsiao-YunTokita, YoshihitoHuang, WeiniTölle, AngelikaHuang, Wen-LiiTolwinski, NicholasHuber, RobertTomar, DhanendraHummel, MichaelTomida, JunyaHung, Jan-jongTonooka, YukiHung, Kuo-HsiangTorkamani, AliHunt, DavidTorres, Adrian GabrielHuotari, TeaTrifonov, VladimirHuppi, KonradTrögl, JosefHur, YoonkangTsuchimoto, DaisukeHüttenhofer, AlexanderTsukahara, ToshifumiHyman, StevenTsutsui, NeilHyun, Sang-HwanTu, MinIbeagha-Awemu, EvelineTuffery-Giraud, SylvieIglesias, AlbertoTurroni, SilviaIkeda, Tatsuya M.Tyagi, AntarikshIlera, Juan CarlosTyrka, MirosławIlinsky, Yury Y.Tzen, J.T.Impey, SorenUchida, ShizukaInamura, KentaroUnver, TurgayIncitti, TaniaUrbanelli, LorenaIngre, CarolineUribe-Convers, SimonInoue, KazushiUrrestarazu, JorgeIorizzo, MassimoUssar, SiegfriedIrie, ToshikazuVähä-Koskela, MarkusIrminger-Finger, IrmgardValdés, Ana ElisaIsaacs, John T.Vallini, GiovanniIshikawa, RyujiVan Dam, PeterIshino, YoshizumiVan Den Berge, MargreetIslam, Salim TimoVan Der Klift, Heleen M.Itzykson, R.Van Der Linde, KarinaIvankovic, AnteVan Der Meer, Jan RoelofIwaizumi, Masakazu G.Van Der Veer, Eric P.Iwasaki, YukaVan Dolah, Frances M.Jackson, MichaelVan Etten, JamieJacobs, EnnoVan Laar, TriciaJacquet, StéphanVan Noorden, CornelisJaligot, EstelleVan Roon, Willeke M.C.James, Euan KevinVankayala, RavirajJarvis, DavidVannucci, LucaJi, Yun-HengVaxillaire, MartineJiang, NingVeedu, RakeshJiao, YinpingVelin, DominiqueJiménez-Conde, JordiVenkataraman, GirishJohansson, PeterVerde, GaetanoJohnson, Warren E.Veronesi, FabioJohnsson, MartinVerschoor, ChrisJones, BrianVicari, Marcelo R.Jones, Peter LawrenceVieira, AlexandreJordao, LuisaVieira, Maria Lúcia CarneiroJuanchich, AmélieViennois, EmilieJuneau, PhilippeVinardell, José M.Jung, Ki-HongVining, KellyJunior, Walter A. RomanViruel, Maria AngelesJunker, KerstinVladimirov, VladimirKageyama, DaisukeVogel, ArndtKaguni, JonVolff, Jean-NicolasKahlert, UlfVolinia, StefanoKalady, Matthew F.Vozdova, MiluseKale, AbhijitVrancken, BramKamisugi, YasukoWachowiak, MattKang, Chang HoWada, JunKao, Gary D.Wagner, RobertKappel, ChristianWalkley, CarlKaranis, PanagiotisWaller, RachelKarijolich, John JosephWalther, WolfgangKataoka, NaoyukiWan, DongshiKatayama, TsutomuWan, JunKato, KeitaWang, BoKatoh, MasaruWang, HsiuyingKavas, MusaWang, JianboKearsey, StephenWang, JiangpingKefalas, PanosWang, Qing-WeiKeiser, Megan S.Wang, RichardKejnovsky, EduardWang, XiaowuKeller, UllrichWang, XinjiangKelly, LauraWang., KaiKennedy, Martin AWasylishen, AmandaKhalyfa, AbdelnabyWebb, Hattie E.Khanna, Kum KumWeber, Bernhard H.F.Khatodia, SurenderWei, FuwenKhatri, BhuwanWei, LeyiKim, Jin-WookWeising, KurtKim, Kyung-MinWeisschuh, NicoleKim, Soo-HyunWerner, BenjaminKim, Young-KookWerr, WolfgangKimball, Rebecca T.White, MalcolmKing, GrahamWhite, PeterKinyamu, HarrietWieser, RotraudKirby, JanineWilliams, Bart O.Kistler, LoganWilson, EricKlähn, StephanWilson, JimKlasson, LisaWilusz, JeffKlengel, TorstenWilusz, Jeremy E.Klimontov, VadimWinternitz, JamieKnoop, VolkerWittstock, UteKober, KordWlodarska, IwonaKoblmüller, StephanWold, MarcKobor, Michael S.Wold, William S. M.Koenekoop, RobertWoldemichael, BisratKoh, Gar YeeWolf, ElmarKolano, BozenaWolf, Paul G.Kolchanov, Nikolay A.Wolfrum, UweKolialexi, AggelikiWon, HyosigKomorowski, JanWood, JamieKondo, HiroyukiWoting, AnniKontogiannatos, DimitriosWu, DiKorsching, Sigrun I.Wu, GuangKosova, KlaraWu, HongjuKothe, UteWu, HuiKotterman, Melissa A.Wu, Lawrence Shih HsinKouvelis, Vassili N.Wu, LiyouKovács, Ákos T.Wu, TongbinKovalovsky, DamianWürtz, SvenKozaki, AkikoXiao, YuguoKozma, Chahira C.Xiong, LanKraikivski, PavelXu, WenyanKrupovic, MartXu, YongjieKschischo, MaikYagüe, ErnestoKu, ChuanYan, ShunpingKubo, NakaoYang, Chin-YingKubota, TakeoYang, ShihuiKuhlmann, MarkusYang, ShulinKukekova, Anna VYang, Wan-XiKummola, LauraYang, ZaijunKunej, TanjaYatera, KazuhiroKuntner, MatjažYatsenko, Alexander N.Kuroiwa, AsatoYe, ZhibiaoKwak, ShinYi, BinLachman, HerbertYin, YanBinLacroix, BenoitYokoi, ShujiLagali, PamelaYoshida, ShuheiLai, Poh SanYoshiyama, HironoriLalueza-Fox, CarlesYoung, Ken H.Lange, LeslieYu, GangLargo, RaquelYu, Jennifer S.Lari, MartinaYu, Yi-TaoLarijani, ManiYue, FengLarson, GregerZacharias, MartinLas Peñas, María LauraZachariou, ZachariasLaSalle, JanineZanin, LauraLaurençon, AnneZegerman, PhilipLautru, SylvieZhang, FengLazarevic, VladimirZhang, JibinLe Hir, RozennZhang, JingjingLebeda, IevgenZhang, LixinLeclercq, AlexandreZhang, LuboLee, Kin Wah TerenceZhang, MeipingLee, SanghyeobZhang, QingpengLeffak, MichaelZhang, QiongLehmann, Alan R.Zhang, XuLehmann, UlrichZhang, YixiangLeon, JavierZhang, ZhiyongLetarov, Andrey V.Zhao, HanLevesque, CelineZhao, QunLezza, AngelaZheng, GuopingLi, DefangZhou, JunLi, GuotianZhou, ZhixiongLi, Lijia LiZhu, ZhouLi, QuanziZima, JanLi, YanjieZippo, AlessioLi, YinZou, QuanLi, YouguoZou, YueLiang, HaiyingZoupa, MariaLiang, QiangrongZsurka, GaborLiao, Pei-Chun


